# In situ identification and G4-PPI-His-Mal-dendrimer-induced reduction of early-stage amyloid aggregates in Alzheimer’s disease transgenic mice using synchrotron-based infrared imaging

**DOI:** 10.1038/s41598-021-96379-4

**Published:** 2021-09-15

**Authors:** Núria Benseny-Cases, Elena Álvarez-Marimon, Ester Aso, Margarita Carmona, Oxana Klementieva, Dietmar Appelhans, Isidre Ferrer, Josep Cladera

**Affiliations:** 1 ALBA Synchrotron Light Source, Carrer de la Llum 2−26, 08290 Cerdanyola del Vallès, Catalonia Spain; 2 Unitat de Biofísica i Centre d’Estudis en Biofísica, Departament de Bioquímica i de Biologia Molecular, Facultat de Medicina, Universitat Autònoma de Barcelona, 08193 Bellaterra, Catalonia Spain; 3 Biomedical Network Research Center of Neurodegenerative Diseases (CIBERNED), University of Barcelona, 08907 Hospitalet de Llobregat, Catalonia Spain; 4 Medical Microspectroscopy, Department of Experimental Medical Science, Lund University, 22180 Lund, Sweden; 5 Leibniz Institute of Polymer Research, Dresden, Hohe Strase 6, 01069 Dresden, Free State of Saxony Germany

**Keywords:** Biochemistry, Biophysics, Chemical biology, Neuroscience

## Abstract

Amyloid plaques composed of Aβ amyloid peptides and neurofibrillary tangles are a pathological hallmark of Alzheimer Disease. In situ identification of early-stage amyloid aggregates in Alzheimer’s disease is relevant for their importance as potential targets for effective drugs. Synchrotron-based infrared imaging is here used to identify early-stage oligomeric/granular aggregated amyloid species in situ in the brain of APP/PS1 transgenic mice for the first time. Also, APP/PS1 mice show fibrillary aggregates at 6 and 12 months. A significant decreased burden of early-stage aggregates and fibrillary aggregates is obtained following treatment with poly(propylene imine) dendrimers with histidine-maltose shell (a neurodegenerative protector) in 6-month-old APP/PS1 mice, thus demonstrating their putative therapeutic properties of in AD models. Identification, localization, and characterization using infrared imaging of these non-fibrillary species in the cerebral cortex at early stages of AD progression in transgenic mice point to their relevance as putative pharmacological targets. No less important, early detection of these structures may be useful in the search for markers for non-invasive diagnostic techniques.

## Introduction

Alzheimer's Disease (AD) is characterized by the presence of senile plaques mainly composed of Aβ peptides and neurofibrillary tangles resulting from aberrant intraneuronal deposition of phosphorylated tau species. However, recent experimental evidence supports the idea that amyloid fibrils are not necessarily toxic whereas toxic species relevant to the onset and progression of the disease are found among a diverse population of on- and off-pathway non-fibrillary intermediates. Although some of these non-fibrillary amyloids and their toxicity have been described in vitro, and different oligomeric species have been isolated from affected brain homogenates^1–3^, the challenge remains regarding their identification in vivo and the time at which they form, presumably long before the appearance of neurological deficits. Whereas powerful imaging techniques such as MRI and PET are being used to detect the presence of fibrillary amyloids, and in some cases, oligomeric species have been targeted^4–8^, the detection of earlier non-fibrillary aggregates and their structural characterization, which would facilitate the design of effective drugs, remain elusive.

Infrared microscopy (µ-FTIR) has been used in the last decade for the in situ study of amyloid deposits^9–13^. In the present work, we have used synchrotron-based µ-FTIR to describe the early formation of non-fibrillar amyloid aggregates in brains of APP/PS1 transgenic mice^14–17^ at 3, 6, and 12 months and to evaluate the effect of poly(propylene imine) dendrimers with histidine-maltose shell (G4-His-Mal dendrimers)^18^ as anti-neurodegenerative agents in mice.

## Results and discussion

Fourier Transform Infrared (FTIR) spectra were measured in the cerebral cortex of 3-, 6-, and 12-month-old APP/PS1 transgenic mice, age-matched wild type (WT) mice and 6-month-old APP/PS1 mice treated with G4-His-Mal dendrimers. Supplementary Figs. S1 and S2 provide two examples of the regions measured in the animal’s cortex and how the detected amyloid deposits co-localize with a positive anti-amyloid antibody label in a contiguous slide of the tissue.

**Figure 1:**
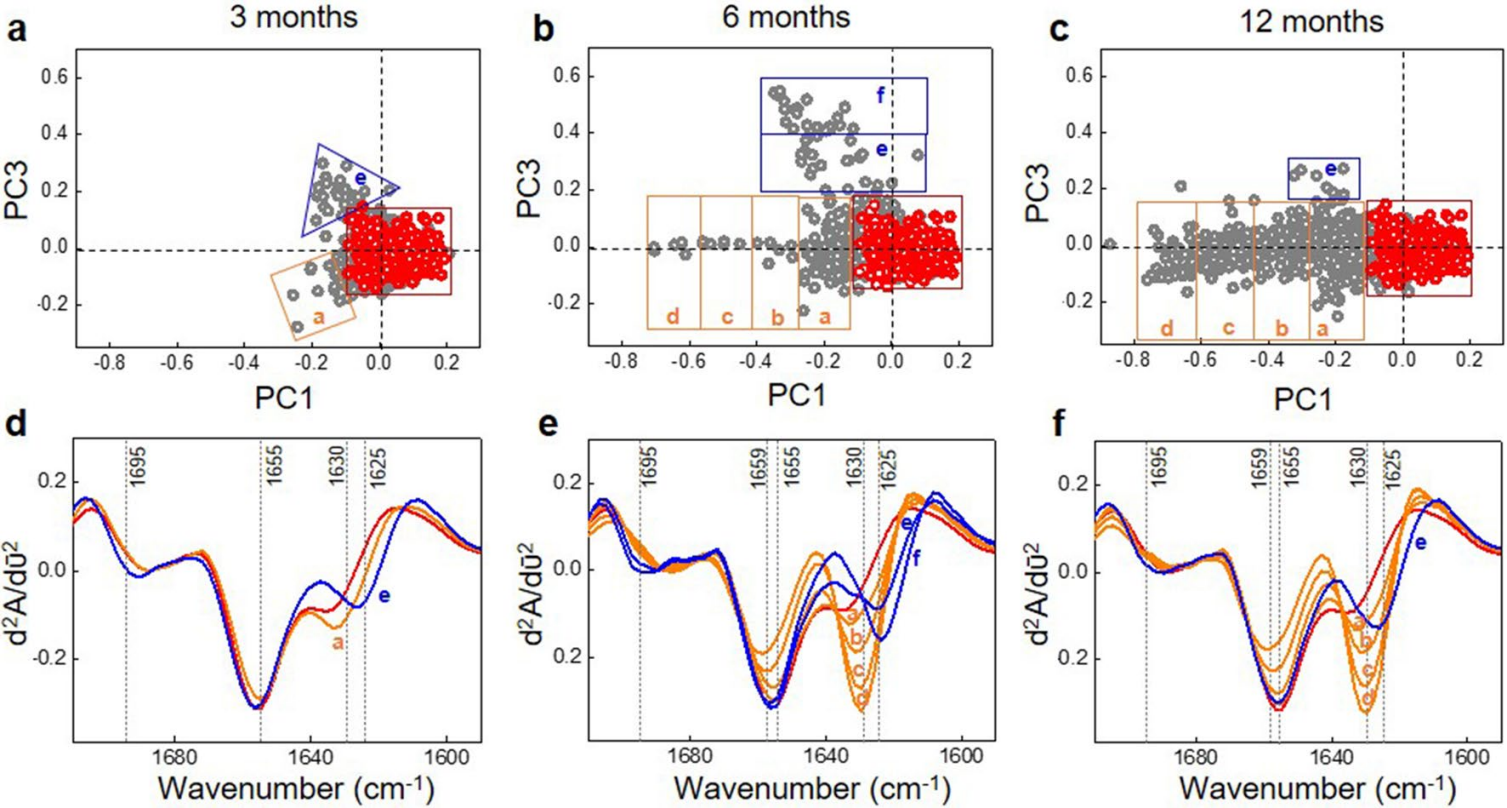
Principal component analysis (PCA) score plots of infrared spectra from brain samples of WT mice and APP/PS1 mice 3 months (**a**), 6 months (**b**) and 12 months (**c**) old. WT data is shown in red and APP data in grey. The loadings corresponding to the PCA scores are shown in Fig. S1 together with the PCA scores of the whole set of data using in this study (APP/PS1 + treated with G4-His-Mal dendrimer). The bottom row shows the normalized second derivative mean spectra of the amide I region calculated from the individual spectra in each outlier segment (**a**–**f**) on the PCA scores, for APP/PS1 mice 3 months (**d**), 6 months (**e**) and 12 months (**f**) old. Colour code: each average derivative spectrum has the same colour as the corresponding coloured segment in which the outlier spectra appear in the top row. In orange, segments (**a**–**d**) in the direction of the negative PC1 axis; in blue, segments (**e**,**f**) in the direction of the positive PC3 component; in red, the average spectrum of the WT data points. Unscrambler X software (CAMO, Oslo, Norway) was used to perform PCA and Origin 9.1 and Microsoft power point 2016 software were used for graphical representation.

The scores graph and the corresponding loadings of the Principal Component Analysis (PCA) of the whole set of data is shown in supplementary Fig. S3. In order to facilitate data visualization and interpretation the score plot of the whole data set has been broken down and the resulting graphs are shown in Figs. 1 and 5 in this section. Representing the principal component 1(PC1) versus the principal component 3 (PC3) has given a score graph in which two set of outliers (points corresponding to spectra outside the region in which the WT spectra appear) are more clearly resolved than in the PC1 vs. PC2 plot (data not shown). According to the loadings graph, the outliers on the PC1 axis differ from the WT spectra in a main feature centred at 1628 cm^−1^, characteristic of peptide/protein fibrillary aggregation. The PC3 loadings show two features centred at 1620 and 1695 cm^−1^, characteristic of non-fibrillary/amorphous/granular (non-fibrillar β-sheet) structures^19–21^.

### Oligomeric/granular (O/G) and fibrillary amyloid aggregates in APP/PS1 mice at 3, 6 and 12 months of age

Figure 1 shows the score plot of the PCA of the amide I region for 3-, 6-, and 12-month-old APP/PS1 represented together with WT data. Since, according to the loadings graph (supplementary Fig. S1) the typical wavenumber for intermolecular fibrillary β-sheet is the dominant feature of PC1, PC1 can be used to classify the spectra as a function of fibrillary amyloid content. In the same way, being PC3 indicative of non-fibrillary β-sheet structures, PC3 values can be used to classify the spectra depending on the presence of oligomeric/granullar β-sheet content. We have done so by segmenting the data points outside the area corresponding to the WT points in two different groups: outliers in the direction of the negative PC1 (segmented in orange in Fig. 1); and outliers in the direction of the positive PC3 (segmented in blue in Fig. 1).

**Figure 2:**
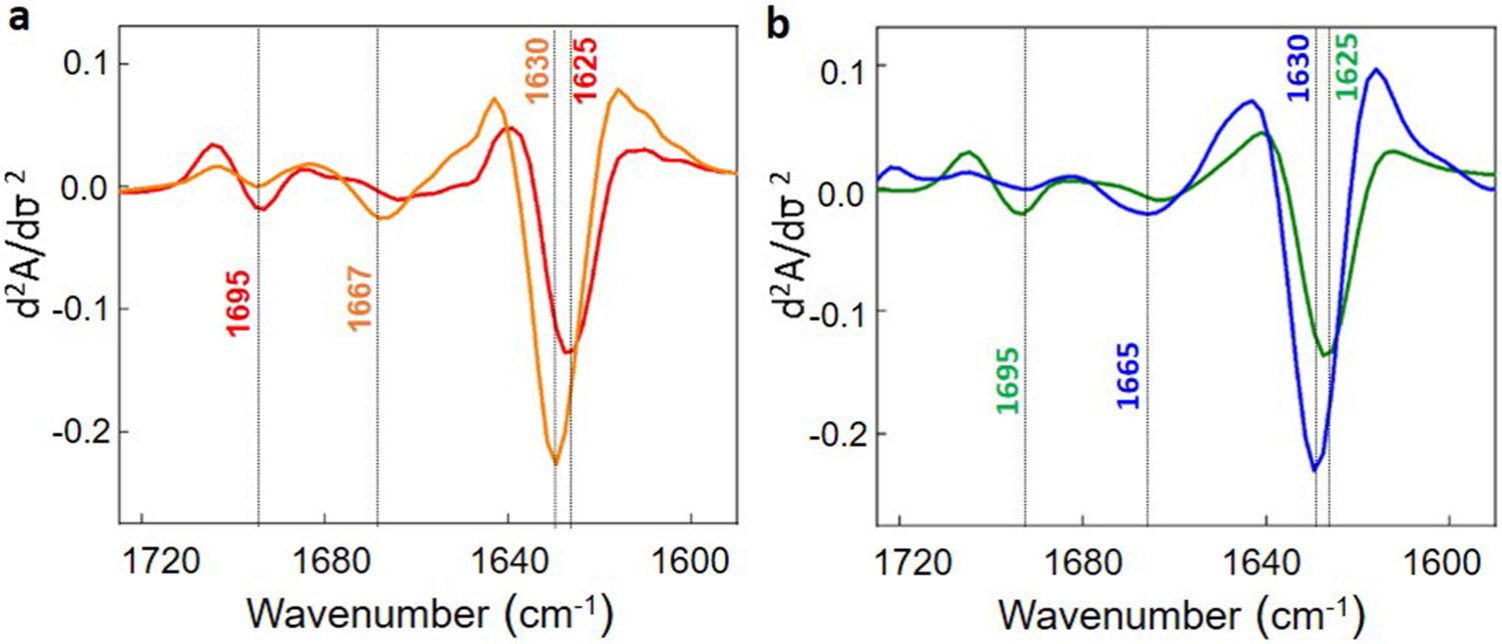
Average second derivative spectra of the amide I region of (**a**) Aβ(1–40) and (**b**) Aβ(1–42) amyloid aggregates generated in vitro: fibrils (orange and blue respectively) generated at pH 7.4 and Aβ granular non-fibrillary aggregates (red and green respectively) generated at pH 5.5. The absorbance spectra were normalized for the amide I area prior to derivation. Origin 9.1 and Microsoft power point 2016 software were used for graphical representation.

In order to further characterize the structural differences between the different groups of outliers the mean of the FTIR spectrum of all data points in each segment in Fig. 1 was calculated and compared with the average spectra of the WT points. The derivative spectra corresponding to the PC1 segments (a–d), depicted in orange in Fig. 1 (bottom panels) show a β-sheet fibrillary aggregation band at 1630 cm^−1^ (consistent with the 1628 cm^−1^ feature detected in the PC1 loadings in Figure S3), which intensity increases as the PC1 value becomes more negative, together with a shift of the minimum at 1655 cm^−1^ towards 1659 cm^−1^. Fibrillary structures can therefore be characterized by the presence of these two features in the second derivative spectra: a band at 1630 cm^−1^ and an increase in intensity at 1659 cm^−1^. The derivative spectra corresponding to the PC3 segments (e,f), depicted in blue, show a band at 1625 cm^−1^ (this band represents a shift with respect to the 1630 cm^−1^ band of the fibrillary structures and is compatible with the shifted 1620 cm^−1^ feature in the PC3 loadings in Figure S3) and a band centred at 1695 cm^−1^ (the same wavelength observed in the PC3 loadings), both characteristic of a non-fibrillar β-sheet structure. Comparison of these spectral features with the FTIR (second derivative) spectra of known aggregated species generated in vitro, shown in Fig. 2, permits to clearly identify the aggregated species detected in APP/PS1 mice brains as oligomeric/granular aggregates (O/G, β structures of non-fibrillary nature, with a high frequency component, characteristic of non-fibrillary β sheets) and fibrillary aggregates. The biophysical analysis of these in vitro aggregates is displayed in Fig. S4, showing the morphology of the fibrils and the non-fibrillary aggregates together with the ThT fluorescence kinetics, which is clearly more intense in the presence of fibrils. Such infrared spectral features for non-fibrillary and fibrillary amyloid structures have also been reported in previous in vitro studies^19–21^. It is worth emphasizing that the spectra depicted in Fig. 2 correspond to the amyloid peptide aggregated under incubation conditions that lead to the formation of either fibrillary aggregates (neutral pH) or amorphous/granular aggregates (pH 5.5). The spectral features of the amorphous aggregated species at pH 5.5 turn out to be very similar to those reported by Sarroukh et al.^20^ for oligomeric Aβ (both Aβ(1–40) and Aβ(1–42)). Our own data together with previous studies show that the spectral features of fibrils and oligomeric/amorphous/granular aggregates are very similar for both Aβ(1–40) and Aβ(1–42). This means that infrared spectra cannot be used as a tool to distinguish between oligomers and amorphous aggregates nor to differentiate the aggregates formed by Aβ(1–40) from the ones formed by Aβ(1–42). However, it clearly differentiates fibrillary from non-fibrillary aggregates.

The correspondence between the distinct spectroscopic features and the fibrillary/non-fibrillary structures is proven by the TEM images shown in Fig. S4. In relation to the aggregated structures at pH 5.5, although the TEM images do not permit to deduce any structural detail other than the amorphous/granular aspect, it is clear that the aggregates are non-fibrillary, and besides the large amorphous clumps individual round dots, compatible with big oligomeric structures are normally distinguished. In our previous studies^19^ with Aβ(1–40) we showed that granular (amorphous) aggregates formed at pH 5.5 could be transformed into fibrils by increasing the pH to 7.4 and that this transformation followed the typical sigmoidal kinetics. For this reason, we speculated with the possibility that these granular aggregates could be a source of oligomeric forms given the right conditions (i.e. an increase of the pH). For all these reasons we refer to the amorphous aggregates of granular aspect with the same spectroscopic characteristics as oligomeric structures as O/G aggregates. Aβ(1–40) O/G aggregates are known to be toxic to neuroblastoma cells in culture^19^. Toxicity assays with Aβ(1–42) have shown that this peptide incubated at pH 5.5 is as well toxic (data not shown).

It can be concluded from Fig. 1 that these two types of aggregated structures (fibrillary and O/G non-fibrillary aggregates) start forming in APP/PS1 mice brains at 3 months of age, both types do clearly increase at 6 months and at 12 months a further increase of the fibrillary structures and a decrease of the O/G aggregates is detected.

**Figure 3:**
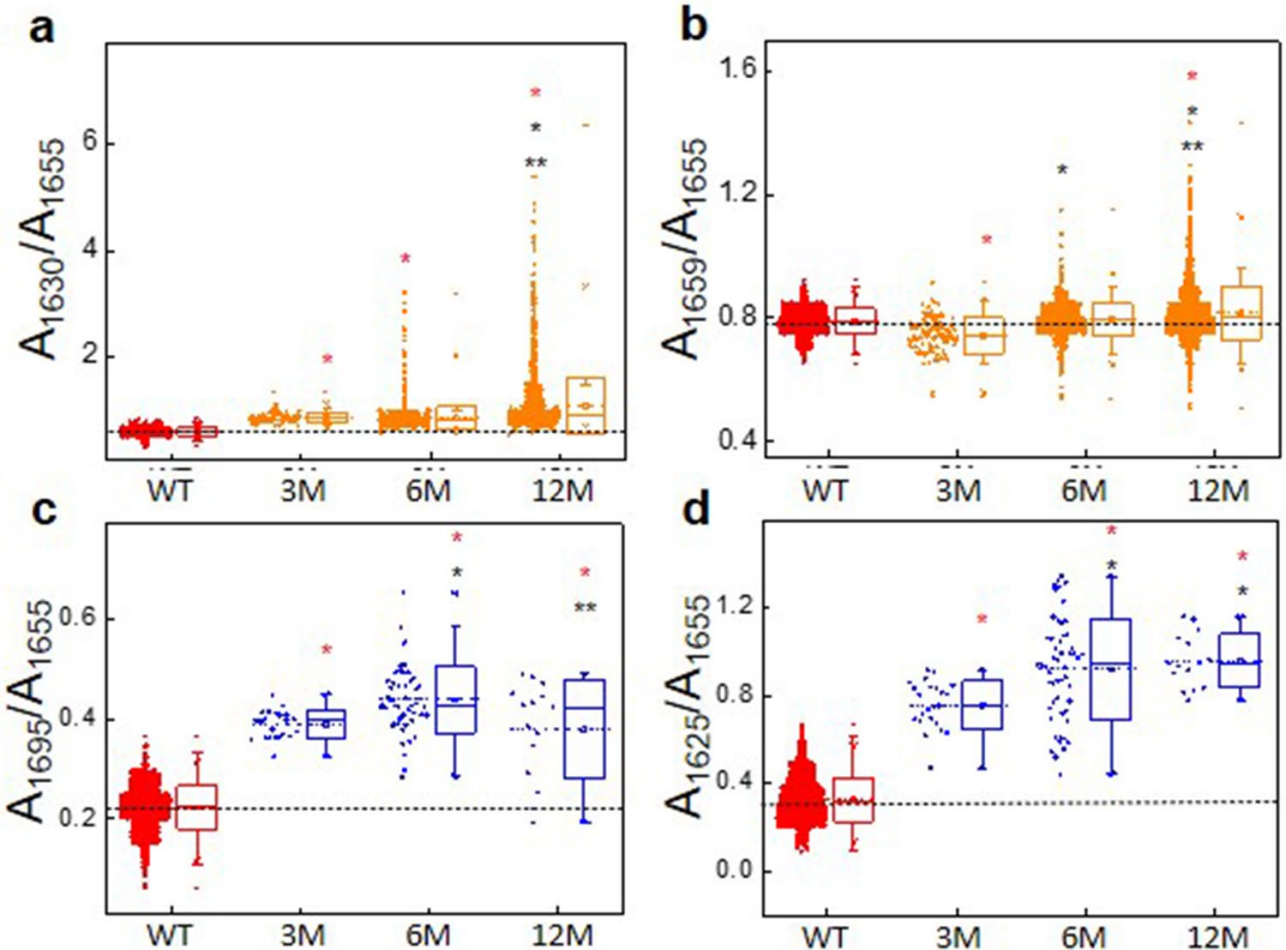
Box plot graphics depicting the absorbance ratio values corresponding to the two different types of aggregated species identified at 3, 6, and 12 months in APP/PS1 and WT mice. Amyloid fibrils (**a**, **b**: the ratios A_1630_/A_1655_ and A_1659_/A_1655_ are the two spectral features of fibrillary structures as explained in “Results and discussion” section) and Oligomeric/granular aggregates (**c**, **d**: the ratios A_1695_/A_1655_ and A_1625_/A_1655_ are the two spectral features of non-fibrillary aggregated structures as explained in “Results and discussion” section). Ratios are calculated from the second derivative spectra of the outliers in each segment (**a**–**f**) in the PCA shown in Fig. 1. Boxplot shows the median (center line), interquartile range (IQR) (box); whiskers represent the most extreme data located no more than 1.5xIQR from the edge of the box, and outliers are the points outside this range. T-test is used for comparison with WT (red*), 12 month mice (*), and between 3 and 6 month mice (**) at p < 0.002. Origin 9.1 and Microsoft power point 2016 software were used for graphical representation.

These results, showing the evolution of both types of amyloid aggregates with mice age, can be formulated as well in terms of the differences in the values of the spectroscopic ratios reported in Fig. 3. For all the ratios, the main band of the WT spectra at 1655 cm^−1^ was taken as the reference band against which the intensity of the characteristic bands of the different aggregated species was measured. The ratios A_1630_/A_1655_ and A_1659_/A_1655_ were taken as the spectroscopic signature of fibrillary aggregates, and the ratios A_1695_/A_1655_ and A_1625_/A_1655_ as the spectroscopic signature of the O/G aggregates.

Experimental points with values of the ratios at A_1630_/A_1655_ and A_1659_/A_1655_ (fibrillary structures) higher than the values calculated for the WT are clearly more abundant at 6 and 12 months and their abundance increase with age (Fig. 3a,b).

Figure 3c,d, shows that the abundance of O/G non-fibrillary structures is significantly increased in APP/PS1 compared to WT (experimental points with a value of the ratios A_1695_/A_1655_ and A_1625_/A_1655_ higher than the values calculated for the wild type), and they are more abundant at 3 and 6 months than at 12 months.

**Figure 4:**
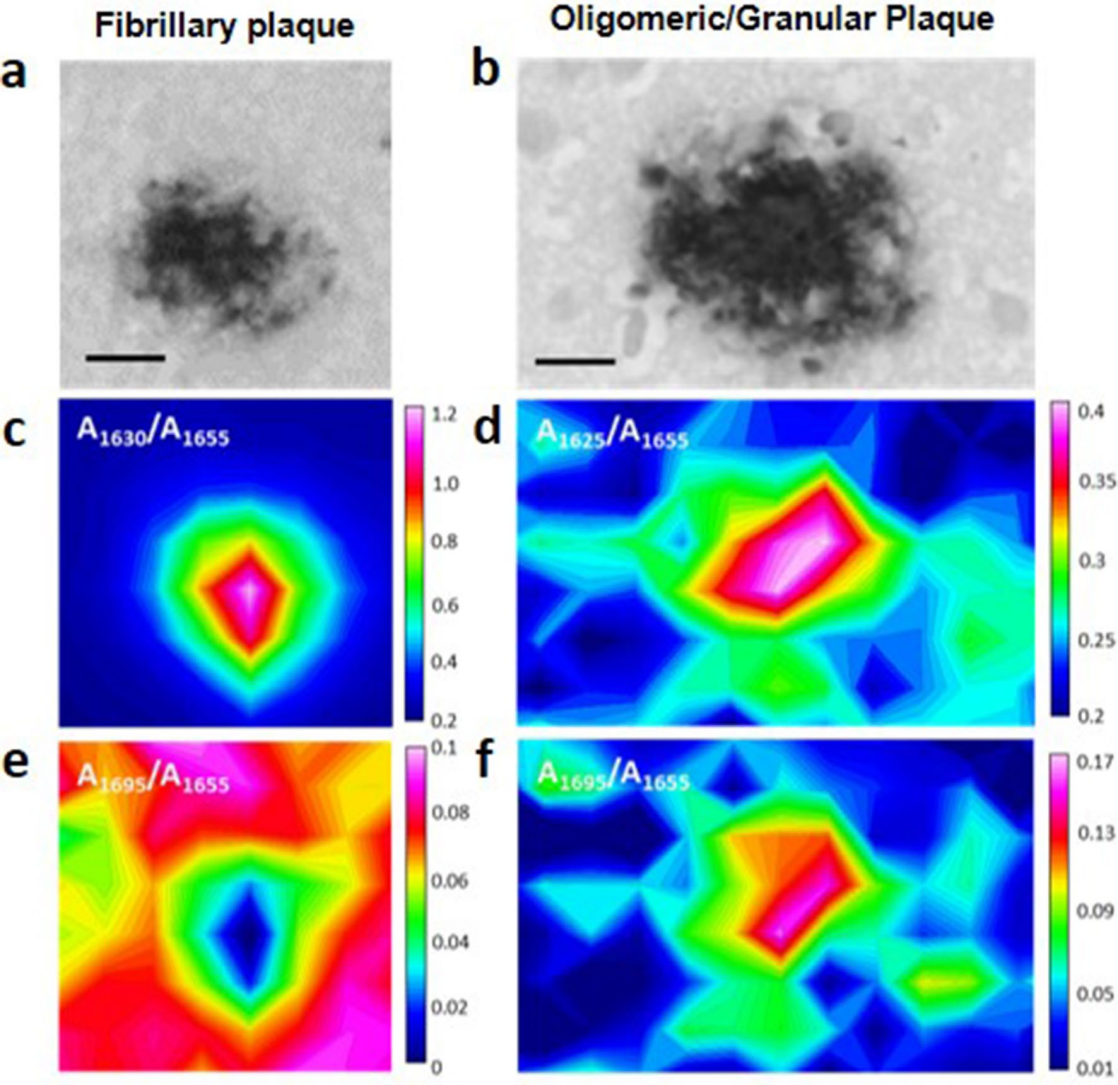
Representative infrared and visible maps of a fibrillary plaque (left panels) and an Oligomeric/Granullar plaque (right panels). (Top row) Images in the visible region of the plaque labeled with an antiamyloid antibody in a contiguous brain slide; (Midle row) absorbance ratios corresponding to fibrillary β-sheet amyloid aggregation; (Bottom row) absorbance ratios corresponding to antiparallel β-sheet aggregation distribution. Ratios were calculated from the second derivative spectra. Scale bar = 10 μm. Opus 7.5 (Bruker) software was used for the maps representation.

Since µ-FTIR makes possible the representation of the spatial distribution of the detected chemical species, the different types of amyloid aggregates (fibrillary, non-fibrillary O/G) can be visualized in the form of infrared maps using the corresponding absorbance ratios, as illustrated in Fig. 4. In the figure, the infrared maps are shown together with the corresponding image obtained in the visible region of a contiguous brain slide labelled with a fluorescent amyloid antibody. A more detailed illustration of the co-localization of the infrared regions in which the infrared spectra have been measured, with the anti-amyloid positively labelled plaques using a contiguous tissue slide is given in supplementary Figs. S1 and S2.

### Effect of G4-His-Mal dendrimers on amyloid aggregation in APP/PS1 mice brains

**Figure 5:**
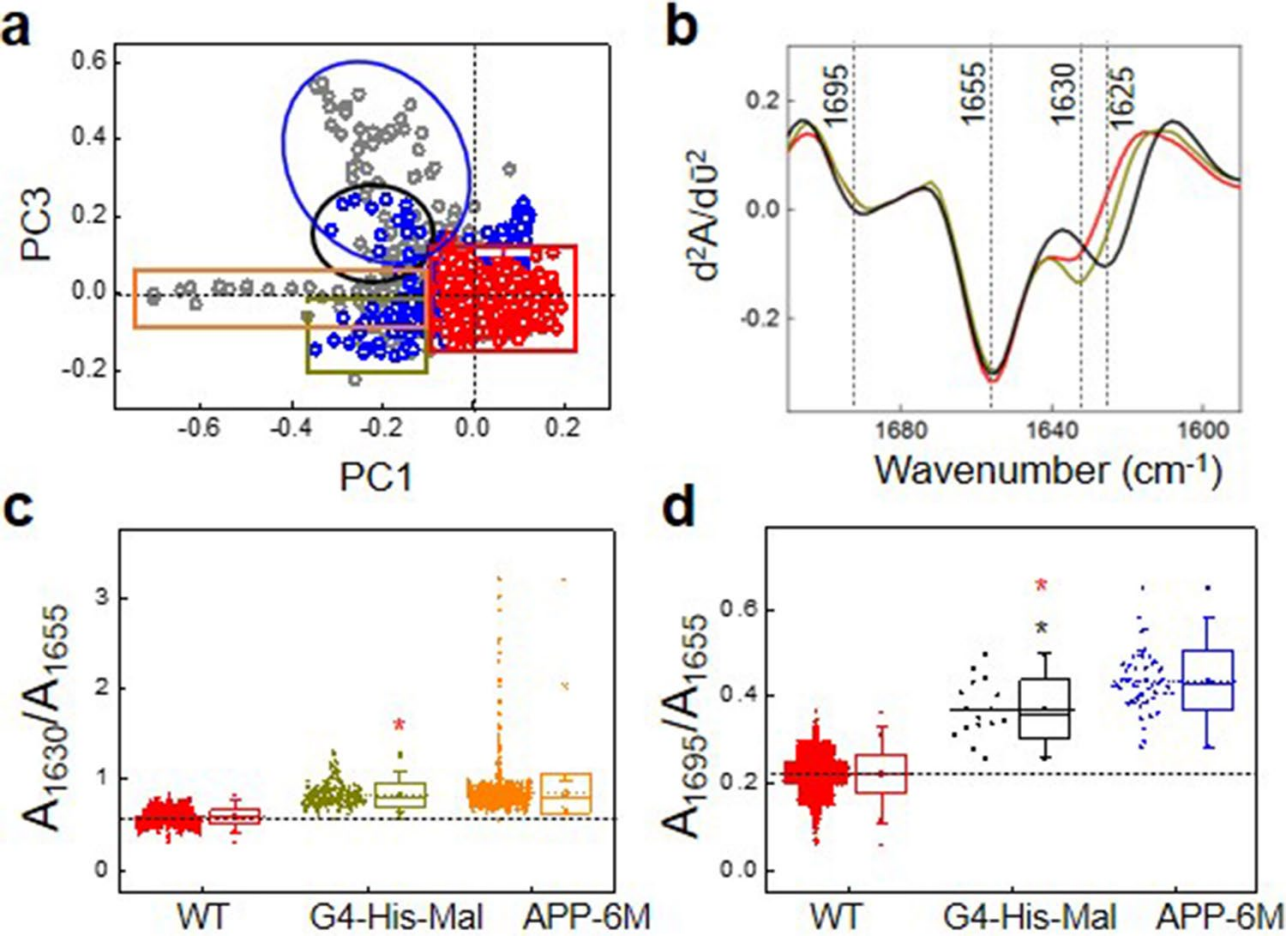
(**a**) Scores of the Principal component analysis (PCA) using the second derivative of the amide I region of infrared spectra showing data from WT mice, APP/PS1 mice 6 months old and APP/PS1 6 months old treated with G4-His-Mal dendrimers. WT data is shown in red, APP/PS1 data in grey and APP/PS1 treated with G4-His-Mal dendrimers in blue. The loadings corresponding to the PCA scores corresponding to the mice treated with G4-His-Mal- dendrimers are depicted in Fig. S1 together with the PCA scores of the whole set of data used in this study; (**b**) normalized mean second derivative of the amide I region calculated from the individual spectra of the data points within the colour circle/rectangle in the outlier regions of the PCA scores. The bottom row shows the box plot graphics depicting the absorbance ratio values corresponding to the two different types of aggregated species calculated from the second derivative spectra of the data points within the colour circle/rectangle in the outlier regions of the PCA scores. Fibrillary aggregates are shown in (**c**) and oligomeric/granular aggregates in (**d**). Boxplot shows the median (center line), interquartile range (IQR) (box); whiskers represent the most extreme data located no more than 1.5xIQR from the edge of the box, and outliers are the points outside this range. T-test is used for comparison with WT (red*) and 6 months mice (*black) at p < 0.002. Colour code: each set of point in the box plots has the same colour as the circle with which the data points from which the ratios have been marked in the scores plot. Unscrambler X software (CAMO, Oslo, Norway) was used to perform PCA and Origin 9.1 and Microsoft power point 2016 software were used for graphical representation.

G4-His-Mal dendrimers are biocompatible globular branched polymers containing maltose and histidine on their surface with potential anti-neurodegenerative properties. Using µ-FTIR we have analysed the effect of G4-His-Mal dendrimers on the aggregated species in the brains of 6-month-old APP/PS1 mice. For these experiments 6-month-old APP/PS1 mice were chosen as they showed significant memory impairment as revealed in the V-maze^17^. The µ-FTIR results clearly show (Fig. 5) that the treatment of APP/PS1 intranasally with a dose of 5 µg per day of G4-His-Mal dendrimers results in reduced formation of early (O/G) amyloid aggregates and that the amount of fibrillary β-sheet is decreased in number and intensity compared to APP/PS1 mice of the same age.

Recently, our group has proven the neuroprotective potential of G4-His-Mal dendrimers, demonstrating that its intranasal administration protects APP/PS1 mice from synapse and memory impairment^18^. This work by Aso et al. showed that G4-His-Mal dendrimers have a significantly improved biocompatibility and the ability to effectively cross the blood brain barrier. Other previously assayed glycodendrimers did not show the capacity to prevent the neurodegenerative effects in transgenic mice. The work by Aso et al. showed as well that G4-His-Mal dendrimers have the capacity to interact, in vitro, with the amyloid peptide, interfering with the nucleation-dependent polymerization process and resulting in a slowing down of the whole reaction and a clear decrease of the amount of ThT-positive fibrils formed at the end of the process. The fibrils which still form show, moreover, a different morphology and appear under the electron microscope as ‘clamped fibrils’, a morphology that has been observed as well previously as a result of the interaction of other (non-maltose) glycodendrimers with the amyloid peptide^22,23^.

Although in the same work Aso et al.^18^, using OC antibodies seemed to observe an increase in Aβ fibrils^18^ the results from the present paper clearly show that the formation of fibrils is reduced. These results could be compatible with the before-mentioned in vitro observation that the interaction of G4-His-Mal dendrimers reduces the formation of ThT-positive amyloid fibrils. However, at this point, we do not have enough data to presume any mechanistic explanation of the molecular interactions by which the action of G4-His-Mal dendrimers result in such a reduced fibril and O/G aggregates formation in the mice affected brains. Besides a direct interaction with the amyloid peptides (or peptide aggregate deposits) one must consider the possibility of indirect effects such as the ones that could be derived from potential ability of the histidine-decorated dendrimer shells acting as metal cation chelators and modulating in this way the influence that copper ions may have for amyloid aggregation and toxicity and the relevance of tissue oxidative processes for the onset and development of the pathology. New research following these avenues, as for example the investigation of the effect of G4-His-Mal dendrimers on the levels of cationic metals in the treated mice brains using synchrotron X-ray fluorescence techniques will be needed. This type of studies may yield results that could permit to gain insight on the molecular mechanisms that could explain the reduction in fibrillization and O/G oligomer formation described in the present work and the neuroprotective effects.

In a previous paper, Klementieva et al.^24^ described the formation of pre-plaque non-fibrillary amyloid structures in Tg19959 transgenic mice. In the present study, we have confirmed the presence of non-fibrillary structures in APP/PS1 mice and have characterized the structure of these aggregates in more detail. Moreover, we show that treatment with G4-His-Mal dendrimers reduces fibrillary aggregation and, importantly, the formation of non-fibrillary (O/G) aggregates.

## Conclusion

Altogether, our results show that non-fibrillary aggregated species (O/G) are abundant in the cerebral cortex at an early stage of disease progression in APP/PS1 transgenic mice. Moreover, the levels of such non-fibrillary structures (and the aggregation level in general) are severely reduced in APP/PS1 mice treated with the spherical dendritic scaffold of H-bond- and ionic interaction-active G4-His-Mal dendrimers.

These non-fibrillary aggregated forms have previously been described in a number of in vitro studies including the formation of synthetic Aβ(1–40) amorphous/granular toxic aggregates at pH 5.5 and in the presence of metal ions and of unordered structures in amyloid aggregates, the occurrence of oligomeric species containing non-fibrillary β structures, and the purification of different types of oligomers such as Aβ*56 from brain homogenates^19–21,25–27^.

Identification, localization, and further characterization of these non-fibrillary species in the cerebral cortex at early stages of AD progression in transgenic mice point to their relevance as putative pharmacological targets. Successful decreased deposition of these species following treatment with G4-His-Mal dendrimers buoys further research in this line. No less important, early detection of these structures may be useful in the search for markers for non-invasive diagnostic techniques.

## Materials and methods

### Animals

All animal procedures were conducted according to the European Union directive 2010/63/EU and approved by the ethics committee of the University of Barcelona. APP/PS1 transgenic mice express a chimeric mouse/human APP (Mo/HuAPP695swe: APP Swedish mutation) and a mutant human presenilin 1 (PS1-dE9); the included Swedish mutation (K595N/M596L) elevates the amount of β-amyloid, and the mutant PS1 allele accelerates the β-amyloid deposition rate as well as exacerbating pathological severity^17^. APP/PS1 transgenic mice and wild-type (WT) littermates were used for the experiments. The human mutated forms APPswe and PS1dE9 were developed by co-injection of the two transgene constructs [Mo/Hu "humanized" chimeric APP695 harbouring the Swedish (K594M/N595L) mutation and exon-9-deleted PS1] delivered by mouse prion promoter into pronuclei with a single genomic insertion site, resulting in the two transgenes being transmitted as a single Mendelian locus. To homogenize the genetic background of mice, the first heterozygous generation was bred for at least 15 generations on a C57BL6J background, with selection for the double mutant transgenes at each generation. Afterward, heterozygote/WT mating produced WT and double-transgenic APP/PS1 littermates for subsequent experiments.

To screen for the presence of the transgenes, genomic DNA was isolated from 1 cm tail clips and genotyped with the polymerase chain reaction (PCR) technique using the PCR conditions proposed by Jackson Laboratory. For the experiment, animals were maintained under standard animal housing conditions in a 12-h dark–light cycle with free access to food and water. The experiments were carried out in compliance with the ARRIVE guidelines.

### Preparation of mice brain tissue for synchrotron FTIR mapping

WT and APP/PS1 mice aged 3, 6, and 12 months (n = 3–4 per time point) were deeply anaesthetized by intraperitoneal injection (0.2 mL/10 g body weight) with a mixture of ketamine (100 mg/kg) and xylazine (20 mg/kg) prior to intracardiac perfusion of 4% paraformaldehyde delivered with a peristaltic pump at 19 mL/min for 5 min. Brains were removed and post-fixed overnight at 4 °C in the same fixative solution. Tissue samples were embedded in paraffin, and coronal sections 8 μm thick were cut on a microtome and mounted on CaF_2_ windows (Crystran, U.K).

### Synthesis of poly(propylene imine) maltose-histidine (G4His-Mal) dendrimers

Synthesis of G4HisMal was performed as in Aso et al.^18^. Briefly, poly(propylene imine) dendrimers of the 4th generation were modified with His (G4-His), and then G4-His was modified with maltose (G4-His-Mal). The whole conversion process was carried out in argon protection atmosphere*.* The structure of G4-His-Mal dendrimers is depicted in supplementary Fig. S5.

### G4-His-Mal dendrimers treatment

The experiments were carried out on male APP/PS1 and wild-type mice. At the age of 3 months, animals were randomly divided as follows: 7 transgenic and 6 WT mice received intranasally a dose of 5 μg per day of G4-His-Mal dendrimer (5 μL of 1 mg/mL solution); 5 transgenic and 6 WT mice received the same volume of PBS (5 μL) until animals reached the age of 6 months.

### Preparation of Aβ(1–40) aggregates in vitro

Aβ(1–40) peptide [DAEFRHDSGYEVHHQKLVFFAEDVGSNKGAIIGLMVGGVV] was purchased from JPT (Germany) with Cl^−^ as a counterion. The peptide (500 μM) was dissolved in 10 mM PBS buffer with 0.04% NH_3_ at pH 12 (pH adjusted using NaOH) and sonicated for 30 s to ensure that it was in monomeric condition as described in Benseny-Cases et al.^19^. The stock solutions were kept at − 80 °C until use. The stock solutions were diluted at 200 μM in PBS buffer and the pH was adjusted to pH 7.4 (to trigger amyloid fibril formation) and to pH 5.5 (to trigger amyloid amorphous aggregates formation) using HCl and were then incubated overnight and mixed at 200 rpm and 37 °C. After incubation, in vitro aggregates were dried directly on CaF_2_ windows.

### SR-FTIR microspectroscopy and data acquisition

SR-μFTIR was performed at the MIRAS beamline at ALBA synchrotron (Catalonia, Spain), using a Hyperion 3000 Microscope that was equipped with a 36× magnification objective coupled to a Vertex 70 spectrometer (Bruker). The measuring range was 650–4000 cm^−1^ and the spectrum collection was carried out in transmission mode at 4 cm^−1^ resolution, 10 μm × 10 μm aperture dimensions, and co-added from 64 to 128 scans. Zero filling was performed with fast Fourier transform (FFT) so that in the final spectra there was one point every 2 cm^−1^. Background spectra were collected from a clean area of the CaF_2_ window every 10 min. Mercury–cadmium–telluride (MCT) detector was used and the microscope and spectrometer were continuously purged with nitrogen gas.

### Fourier transform infrared (FTIR) spectrum analysis

Fourier transform infrared (FTIR) spectra from the different maps, and the independent spectra of amyloid aggregates were analyzed with Opus 7.5 (Bruker) software. Atmospheric compensation was applied to the spectra to remove water vapour and CO_2_ contributions. The spectra exhibiting a low signal-to-noise ratio were eliminated and concave rubberband baseline corrected (RBC) in the range of 3100–1400 cm^−1^ using 64 baseline points and 5 iterations. An example of one set of spectra after and before rubberband corrections is displayed in supplementary Figure S6. Unscrambler X software (CAMO, Oslo, Norway) was used to perform PCA in the data set as in Benseny-Cases et al.^10^. Briefly, PCA analysis was applied on the second derivative of the spectra calculated using a Savitsky-Golay algorithm with a thirteen point filter and a polynomial order of 3. Unit vector normalization was applied after secondary derivation for PCA analysis.

Principal components (PCs) were calculated using the Unscrambler X software (CAMO) Software was also used for the normalized average spectra and the second derivative spectrum calculation. Ratios were calculated over the following peaks representing different protein secondary structures in the Amide I region: 1695 cm^−1^/1655 cm^−1^ associated to 1625 cm^−1^/1655 cm^−1^ for the non-fibrillary (oligomeric, granular aggregates) β-sheet structures (noted as A_1695_/A_1655_ and A_1625_/A1_655_), 1630 cm^−1^/1655 cm^−1^ (noted as A_1630_/A_1655_) and 1659 cm^−1^/1655 cm^−1^ (noted as A_1659_/A_1655_) for the fibrillary β-sheet structures. Origin 9.1 software was used for the ratio calculation and graphical representation.

### Immunohistochemistry

Consecutive 4 µm slices of the tissue samples were embedded in paraffin used for the µFTIR analyses were cut and placed in polyLys treated glass slides. Samples were de-waxed by submerging the samples on xylene (3 × 8 min) and samples were hydrate in 100%, 95%, and 75% ethanol for 2 × 4 min each. De-waxed sections were incubated with 98% formic acid (3 min) and samples were boiled in citrate buffer (20 min) to enhance antigenicity. Then, the endogenous peroxidases were blocked with Peroxidase-Blocking solution (Dako, Denmark) (15 min) and then incubated at 4 °C overnight with the primary antibodies against β-amyloid (Clone 6F/3D)(Dako). Sections were subsequently rinsed and incubated with biotinylated goat anti-mouse and rabbit secondary antibody (Dako), followed by EnVision + System Peroxidase (Dako) and finally with the chromogen diaminobenzidine and H_2_O_2_. Sections were lightly counterstained with hematoxylin. After staining, the sections were dehydrated and cover-slipped for microscopic observation^17^.

## Supplementary Information

Supplementary Information.
