# Supplementary material for: In situ identification and G4-PPI-His-Mal-dendrimer-induced reduction of early-stage amyloid aggregates in Alzheimer’s disease transgenic mice using synchrotron-based infrared imaging
Source: Sci Rep. 2021 Sep 15;11:18368. doi: 10.1038/s41598-021-96379-4 (PMC8443673; doi:10.1038/s41598-021-96379-4)
Supplement: Supplementary file 1 — Supplementary Information. [file 41598_2021_96379_MOESM1_ESM.pdf]

Supplementary Information

In situ identification and G4-PPI-His-Mal-Dendrimer-induced reduction of early-stage amyloid aggregates in Alzheimer's disease transgenic mice using synchrotron-based infrared imaging

Núria Benseny-Cases*[a], Elena Álvarez-Marimon[b], Ester Aso[c], Margarita Carmona[c], Oxana Klementieva[d], Dietmar Appelhans[e], Isidre Ferrer[c] and Josep Cladera*[b]

[a]ALBA Synchrotron Light Source, Carrer de la Llum 2−26, 08290 Cerdanyola del Vallès, Catalonia, Spain.

[b]Unitat de Biofísica i Centre d'Estudis en Biofísica, Departament de Bioquímica i de Biologia Molecular, Facultat de Medicina, Universitat Autònoma de Barcelona, 08193 Bellaterra, Catalonia, Spain.

[c]University of Barcelona; Biomedical Network Research Center of Neurodegenerative Diseases (CIBERNED), 08907 Hospitalet de Llobregat, Catalonia, Spain.

[d]Medical Microspectroscopy, Department of Experimental Medical Science, Lund University, 22180 Lund, Sweden

[e]Leibniz Institute of Polymer Research, Dresden, Hohe Strase 6, D-01069 Dresden, Free State of Saxony, Germany.

*Corresponding authors: nbenseny@cells.es; josep.cladera@uab.cat

Figure S1. Co-localization example for the fibrillary plaque showed in figure 4.
Figure S2. Co-localization example for the O/G plaque showed in figure 4
Figure S3. Principal component analysis (PCA) using the second derivative of the amide I region of infrared spectra from brain samples of WT mice, APP/PS1 mice 3, 6, and 12 months old, APP/PS1 6 month old treated with G4-His-Mal dendrimers.
Figure S4. Biophysical characterisation of the Aβ(1-40) and Aβ(1-42).
Figure S5. Structure of Poly (propyelene imine) maltose-histidine (G4HisMal) dendrimers. Adapted with permission from reference 19.
Figure S6. Spectral correction of some representative data. a) Raw data and b) and spectra after rubberband correction.

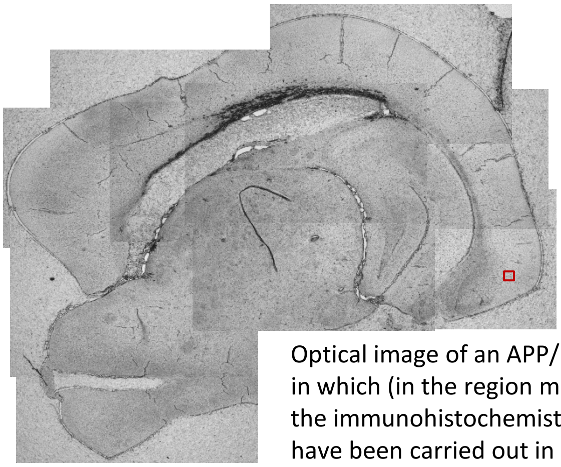

Optical image of an APP/PS1 cortex section
in which (in the region marked with the red circle)
the immunohistochemistry and the IR imaging
have been carried out in contiguous slides

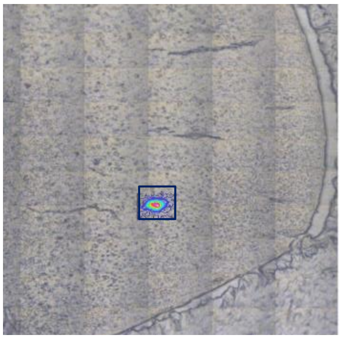

Magnified optical image
in which the with IR map has
been measured

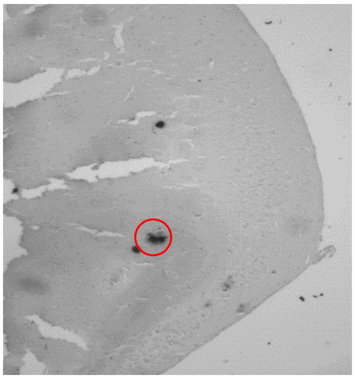

Optical image of contiguous slide
labelled with Aβ antibody

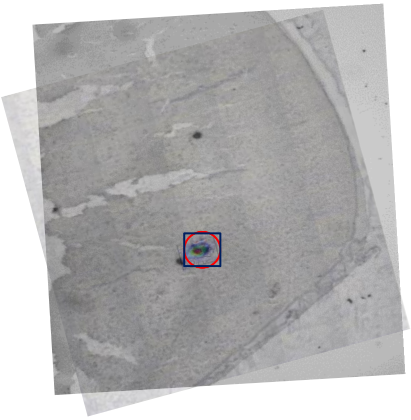

The two optical images superimposed

Figure S1. Co-localization example for the fibrillary plaque showed in figure 4. Microsoft power point 2016 software was used for graphical representation.

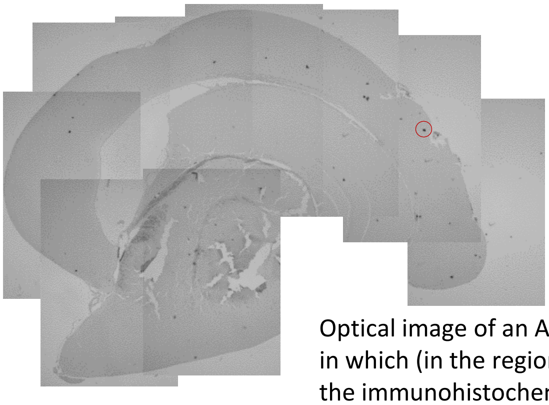

Optical image of an APP/PS1 cortex section
in which (in the region marked with the red circle)
the immunohistochemistry and the IR imaging
have been carried out in contiguous slides

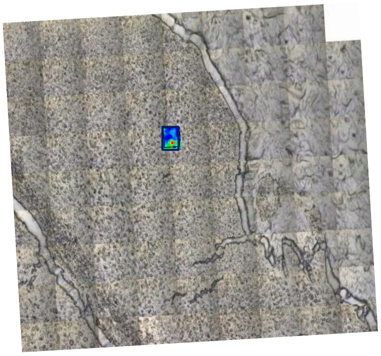

Magnified optical image
in which the with IR map has
been measured

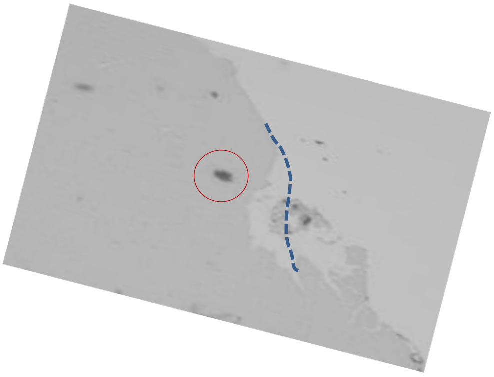

Optical image of contiguous slide
labelled with Aβ antibody

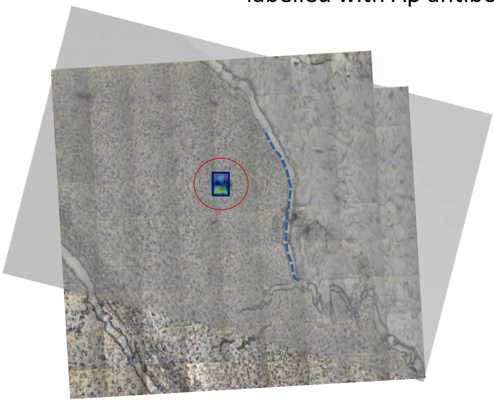

The two optical images superimposed

Figure S2. Co-localization example for the O/G plaque showed in figure 4. Microsoft power point 2016 software was used for graphical representation.

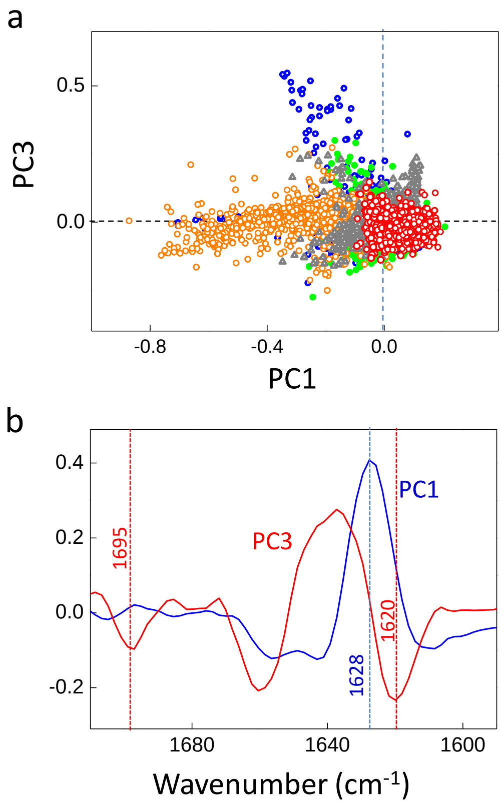

Figure S3. Principal component analysis (PCA) using the second derivative of the amide I region of infrared spectra from brain samples of WT mice, APP/PS1 mice 3, 6, and 12 months old, APP/PS1 6 month old treated with G4-His-Mal dendrimers. (a) Score graph of the PCA analysis and (b) PC1 and PC3 loadings. Color code: WT in red, APP/PS1 3 months in light green, 6 months in blue, 12 months in orange, APP/PS1 6 month old treated with G4-His-Mal dendrimers in grey. Unscrambler X software (CAMO, Oslo, Norway) was used to perform PCA and Origin 9.1 and Microsoft power point 2016 software were used for graphical representation.

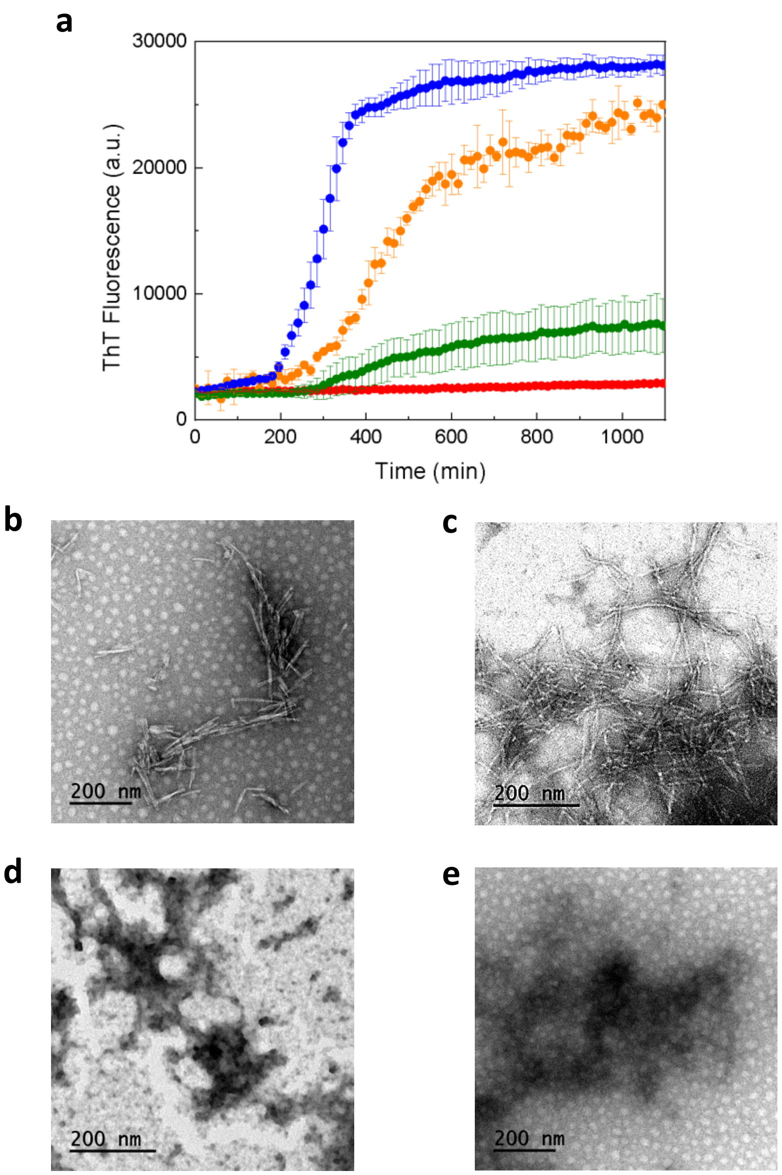

Figure S4. Biophysical characterisation of the Aβ(1-40) and Aβ(1-42).a) ThT aggregation kinetics at 10 µM final concentration and 20 µM of ThT at 20 mM Sodium phosphate buffer. Triplicates of each condition were measured. Colour code: Aβ(1-40) at pH7.4 in orange, Aβ(1-40) at pH5.5 in red, Aβ(1-42) at pH7.4 in blue, Aβ(1-42) at pH5.5 in green. b) Electron microscopy of Aβ(1-40) at pH7.4; c) Aβ(1-42) at pH7.4; d) Aβ(1-40) at pH5.5; e) Aβ(1-42) at pH5.5. Origin 9.1 and Microsoft power point 2016 software were used for graphical representation.

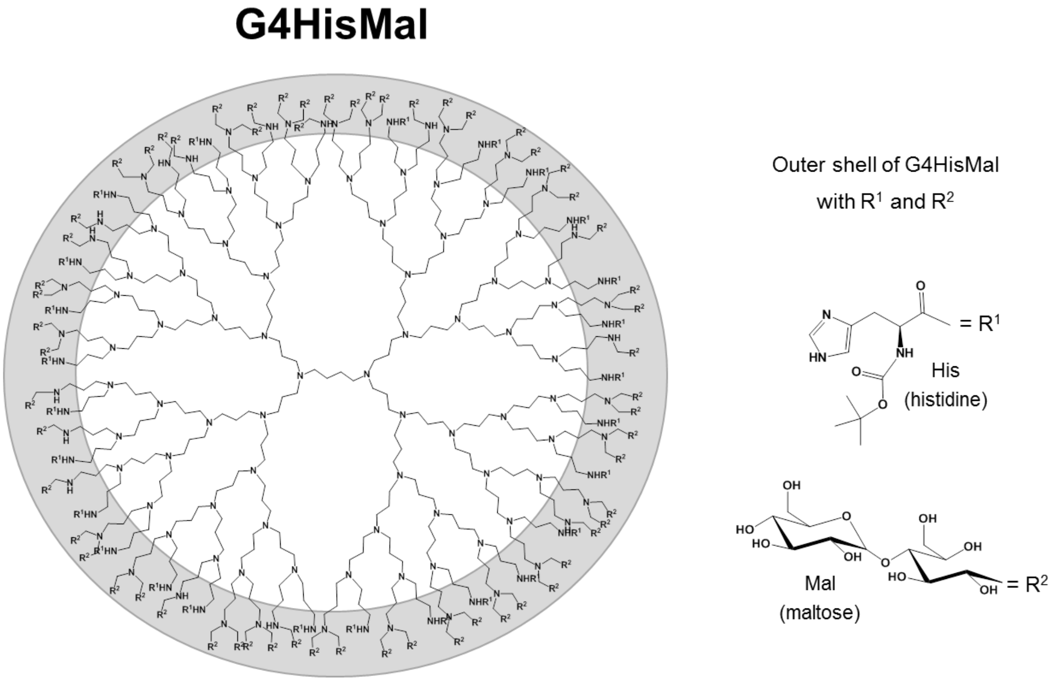

Figure S5. Structure of Poly (propyelene imine) maltose-histidine (G4HisMal) dendrimers. Adapted with permission from reference 19. Microsoft power point 2016 software was used for graphical representation.

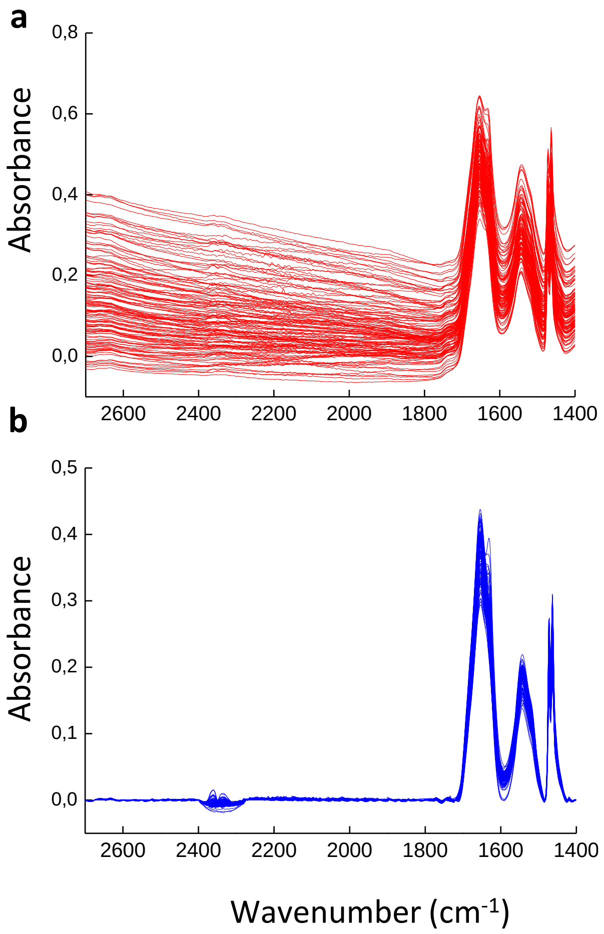

Figure S6. Spectral correction of some representative data. a) Raw data and b) and spectra after rubberband correction. Origin 9.1 and Microsoft power point 2016 software were used for graphical representation.
